# Indian Superstition

**Published:** 1875-09

**Authors:** 


### INDIAN SUPERSTITION.

The Indian's fear of a human corpse, the fear of being brought in contact with it, or with anything that has touched it, is absolutely pitiable. A little girl connected with the Oregon mission school died June 14, and it was resolved to give her a Christian burial. The Indian mode is to remove the corpse off the premises instantly after the breath leaves the body, and as they have a superstition that if carried through the door of the house, the first person that passes through the door is doomed to die soon afterward, the general custom is to make an opening through the side of the house through which the body is passed out, and then the opening is closed. This makes the exit of the dead possible without endangering the life of the other inmates. But the body of this child was taken in charge by the agent. She was properly laid out immediately after death, and none of the Indians were allowed to interfere while a good coffin was made by the reservation carpenter. A grave was dug at a point near where the school is kept, the funeral was appointed at 3 o'clock, and all the Indians invited to attend. The distance from the mission to the grave is two miles, and it was necessary to carry the coffin in a canoe. The head chief of the tribe, a man of more intelligence and more manhood than any other Indian in the tribe, was ordered to take the corpse into the canoe, with the mother of the deceased, and carry them to the place of burial. The chief did not object to do this. He never objects to any order of the agent, however disagreeable. He is always prompt to obey any request. The coffin was brought to the beach, some hundred yards from the grave, where the school and the agent's family met it in procession. The purpose was to have the Indian boys at the school act as bearers. This they were all afraid to do. But it was a point to gain to induce some of them to do this service. Out of twelve boys, four were induced by persuasion, amounting almost to compulsion, to put a hand to the bier and bear the coffin to the grave. The lid was then lifted, and all the school children brought near to view the face of the corpse, an act that caused the children much fear, and to the older Indians it seemed horrible. The school thus standing around the grave sang the familiar school song, "Rest for the Weary," and after prayer, offered by the agent, the other sweet hymn, "There is a Beautiful Land on High," when the coffin was lowered and buried. But the fact of most significant import remains yet to be stated, a fact that reveals the deep-seated superstition of those people. Do you think that canoe in which the chief conveyed that corpse was ever to be used again? By no means. Having been the receptacle of a coffin containing a human corpse, it must never again be used as a vehicle for the living. And the owner demolished it on the spot, in no spirit of anger toward the agent who had ordered a duty evidently revolting to his feelings, but more in the spirit of a willing sacrifice upon the alter of his superstition.

